# Genetic and Pharmacological Blockade of Sigma-1 Receptors Attenuates Inflammation-Associated Hypersensitivity during Acute Colitis in CD1 Mice

**DOI:** 10.3390/biomedicines11102758

**Published:** 2023-10-12

**Authors:** Sergio López-Estévez, Mònica Aguilera, Georgia Gris, Beatriz de la Puente, Alicia Carceller, Vicente Martínez

**Affiliations:** 1 Department of Cell Biology, Physiology and Immunology, Universitat Autònoma de Barcelona, 08193 Barcelona, Spain; sergio.lopez.estevez@gmail.com (S.L.-E.);; 2 Neuroscience Institute, Universitat Autònoma de Barcelona, 08193 Barcelona, Spain; 3 Department of Pharmacology, Welab Barcelona, 08028 Barcelona, Spain; ggris@welab.barcelona (G.G.); bdelapuente@welab.barcelona (B.d.l.P.); acarceller@welab.barcelona (A.C.); 4 Centro de Investigación Biomédica en Red de Enfermedades Hepáticas y Digestivas (CIBERehd), Instituto de Salud Carlos III, 28049 Madrid, Spain

**Keywords:** BD1063, colitis, E-52862, hypersensitivity, intestinal inflammation, pain, sigma 1 receptor, visceral pain

## Abstract

Sigma-1 receptors (σ_1_Rs) are implicated in nociception, including pain sensitization, and inflammation. We assessed the role of σ_1_Rs on acute colitis-associated hypersensitivity using both genetic (constitutive knockout) and pharmacological blockade of the receptor. Colitis was induced in CD1 wild-type (WT) and σ_1_R KO mice (exposure to dextran sodium sulfate, 3%). A von Frey test was used to assess referred mechanosensitivity (abdominal and plantar withdrawal responses). The effects of the selective σ_1_R antagonists BD1063 and E-52862 were also assessed in WT animals. The expression of immune and sensory-related markers (RT-qPCR, Western blot) was assessed in the colon and lumbosacral spinal cord. The genetic ablation or pharmacological blockade of σ_1_Rs attenuated acute colonic inflammation in a similar manner. Mechanosensitivity was similar in WT and σ_1_R KO mice before colitis. In WT mice, but not in σ_1_R KO, colitis was associated with the development of referred mechanical hypersensitivity, manifested as a reduction in the withdrawal thresholds to mechanical probing (paw and abdominal wall). In WT mice, BD1063 and E-52862 blocked colitis-associated hypersensitivity. A genotype- and treatment-related differential regulation of sensory-related markers was detected locally (colon) and within the spinal cord. σ_1_Rs are involved in the development of acute intestinal inflammation and its associated referred mechanical hypersensitivity. The selective modulation of sensory-related pathways within the colon and spinal cord might be part of the underlying mechanisms. These observations support the pharmacological use of σ_1_R antagonists for the treatment of intestinal inflammation-induced hypersensitivity.

## 1. Introduction

Visceral pain is a common symptom in inflammatory and functional gastrointestinal (GI) disorders [1,2,3]. In these conditions, multiple factors, including the release of inflammatory mediators combined with a disturbed epithelial barrier function, contribute to the sensitization of peripheral nerve endings within the gut wall, thus resulting in an altered visceral sensory perception and abdominal pain [2,4]. Moreover, visceral pain typically refers to non-visceral somatic structures due to the convergence of visceral and somatic nerve fibers in the same second-order neurons within the dorsal horn of the spinal cord [5,6]. Therefore, states of visceral pain/hypersensitivity are frequently associated with referred somatic hypersensitivity. Taking into account the differences in their anatomical origin and the neurobiological mechanisms that mediate the sensory process, visceral and somatic pain are considered different entities. A comprehensive review of the visceral pain neurobiological substrata, including the pathways and receptors/mediators involved, can be found in [3,5,6]. So far, specific pharmacological treatments against visceral pain, such as pain arising from the GI tract, have not been approved, and clinicians often use the same medications as for somatic pain [3].

In this respect, several studies suggest sigma-1 receptors (σ_1_Rs) as an effective pharmacological target for pain treatment, including visceral pain [7,8,9]. The σ_1_R is a ligand-regulated molecular chaperone with a wide central and peripheral distribution, which has been implicated in a variety of physiological and pathological conditions, including immuno- and neuromodulatory effects related to the modulation of pain mechanisms at either the central or peripheral levels [7,8,9]. Interestingly, several pieces of evidence suggest that the pharmacological agonism or antagonism of σ_1_R does not interfere with the perception of several stimuli in basal conditions [10,11] and, consequently, with normal pain responses. However, under pathological conditions with states of altered pain signaling, such as in some chemical and neuropathic somatic pain models, the genetic or pharmacologic blockade of σ_1_Rs could have a positive impact on sensory mechanisms, leading to the modulation of pain behavior and hypersensitivity [10,12,13]. Following these observations, further studies have validated the pharmacological blockade of σ_1_R as an effective option to treat central and peripheral inflammatory pain [9,14,15,16]. Although these evidences, similar studies related to visceral pain are scarce. In a model of visceral pain induced by the intracolonic injection of capsaicin, and in a model of cyclophosphamide-induced cystitis, the constitutive absence of σ_1_Rs (σ_1_R knockout (KO) mice) or the blockade of the receptor with selective antagonisms resulted in a reduction in the number of pain-related behaviors [17,18]. Following this, we have shown the implications of σ_1_Rs on the development of intestinal inflammation-associated referred hypersensitivity in C57BL76 σ_1_R KO mice [19]. To further expand these observations, the present work aims to assess the potential role of σ_1_Rs in the development of acute intestinal inflammation and inflammation-related hypersensitivity in CD1 mice with dextran sodium sulfate (DSS)-induced acute colitis. For this purpose, two approaches were followed: (i) the genetic blockade of σ_1_Rs based on the constitutive absence of the receptor, using KO mice; and (ii) the pharmacological blockade of σ_1_Rs, using the selective σ_1_R antagonists BD1063 and E-52862. Moreover, to gain insight into the underlying mechanisms associated with σ_1_R-mediated effects, changes (gene and protein expression) in the peripheral (colon) and central (spinal cord) sensory-related markers involved in pain processing and sensitization mechanisms were also characterized.

## 2. Materials and Methods

### 2.1. Animals

Young adult male CD1 mice (n = 96; Crl/CD1 (ICR); Charles River, France) and σ1 receptor knockout CD1 mice (n = 16) [20], 6–7 weeks old at the beginning of the studies, were used. The animals were generated and maintained by Charles River under a contract with Esteve Pharmaceuticals S.A. (Barcelona, Spain). To obtain CD-1 KO mice, homozygous KO mice [18] were backcrossed for 10 generations onto the CD-1 background to reduce to less than 1% the genetic material remaining from the original background [21]. The animals were group-housed in standard polycarbonate cages (4–6 animals per cage, with direct bedding of wood fibrillated fibers) and maintained in conventional conditions in an environmentally controlled room (20–22 °C, 12 h light/dark cycle), with food and water ad libitum, except when receiving DSS. The mice were allowed to acclimatize to the animal facility for at least 1 week before starting the studies. All experiments were performed in accordance with the EU and local regulations and were approved by the Ethical Committee of the Universitat Autònoma de Barcelona (protocols 3039 and 3957) and the Generalitat de Catalunya (protocols 8823 and 9915).

### 2.2. Colitis Induction

A solution of DSS (45 kDa; 3% concentration in normal tap water; TdB Consultancy AB, Uppsala, Sweden) was used to induce colitis. Fresh DSS solutions were prepared daily during the 5-day treatment period. Following this protocol, CD1 mice develop a flare of acute colitis that peaks 7–8 days after starting the exposure to DSS. Similar protocols have been used in previous studies in mice to induce colitis [22,23]. The control mice received normal tap water.

### 2.3. Drugs

The selective σ_1_R antagonists BD1063 (1-[2-(3,4-dichlorophenyl) ethyl]-4-methylpiperazine dihydrochloride) [24] and E-52862 (also named S1RA or MR309; 4-[2-[[5-methyl-1-(2-naphthalenyl)-1H-pyrazol-3-yl]oxy]ethyl] morpholine) [25] were used (Laboratorios Dr. Esteve S.A., Barcelona, Spain). 6-Thioguanine (6-TG) (Sigma-Aldrich, St. Louis, MO, USA) and 5-aminosalicylic acid (mesalazine, 5-ASA) (Cayman Chemical, Ann Arbor, MI, USA) were used as positive controls, given their demonstrated anti-inflammatory activity within the GI tract [26,27]. All drugs were dissolved immediately before use in a 0.5% solution of hydroxypropylmethyl cellulose (HPMC; Sigma-Aldrich) in distilled water. The doses were selected based on previously published data and/or pilot studies in our experimental conditions.

### 2.4. Evaluation of Referred Mechanical Hypersensitivity: Von Frey Test

**Figure 1:**
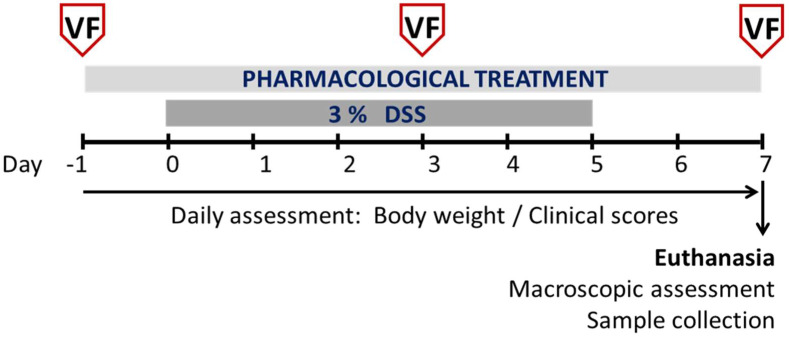
Details of the experimental protocols followed. See Table 1 for details on the treatments applied. VF: von Frey test.

The mechanical sensitivity was determined using the classical von Frey test, following previously published protocols [19]. The animals were placed in compartment enclosures in a test chamber with a framed metal mesh floor through which von Frey monofilaments (North Coast Medical, Inc., Gilroy, CA, USA) were applied. Pain sensitivity was assessed after a 30 min habituation period to the testing environment. Referred pain was determined in two separate body regions, the hind paws and the abdominal wall. When testing the sensitivity of the abdominal wall, the perianal and external genitalia areas were avoided, concentrating the stimulation on the lower and mid-abdomen, as commonly reported in the literature [17,18,28]. Paw sensitivity was quantified by measuring the hind paw withdrawal response to punctate mechanical stimulation, as described elsewhere [10,11]. Pain thresholds were determined using the up-down method paradigm and represent the mechanical stimulus that produces 50% of the maximal response [29,30]. The data were normalized to a baseline measurement (taken as 1), obtained 24 h before starting the experimental procedures (Figure 1). All measurements were performed twice, with a 30–40 min recovery period in between, by two treatment-blinded independent investigators. For each animal, the mean value of the two observations was taken as the measure of pain sensitivity.

### 2.5. Experimental Protocols

In the first study, the WT and σ_1_R KO mice were randomly divided into 2 experimental groups per genotype, receiving either tap water or a solution of 3% DSS during a 5-day period (days 0–5) (Table 1). The animals were euthanized after a 2-day recovery period following DSS exposure (experimental day 7) for the assessment of colitis and the obtention of samples (Figure 1), coinciding with the peak of inflammation [23,31,32]. Throughout the study, their individual body weight, general state, and the presence of clinical signs associated with the development of colitis were assessed on a daily basis. Mechanical sensitivity (von Frey test) was assessed at the beginning of the studies (experimental day −1, as a baseline measure of sensitivity), during DSS exposure (experimental day 3), and at the peak of inflammation (immediately before euthanasia, experimental day 7) (Figure 1).

In the second study, the WT mice were randomly divided into five experimental groups according to the treatment received (Table 1): vehicle (0.5% HPMC in distilled water, 5 mL/kg, po), BD1063 (20 mg/Kg, po, BID), E-52862 (20 mg/Kg, po, BID), 6-TG (2 mg/kg, po, SID), or 5-ASA (50 mg/kg, po, BID). The treatments were applied in a preventive manner starting 1 day before the initiation of the colitis induction protocol and after the baseline measurement of sensitivity. The animals were administered orally once (9:00–10:00 AM) or twice daily (9:00–10:00 AM and 18:00–19:00 PM), as indicated (Table 1). The doses and treatment protocols were based on pilot studies or previous reports showing efficacy in similar experimental conditions. For each treatment group, the animals were randomly divided into 2 subgroups, receiving either tap water or 3% DSS during a 5-day period (experimental days 0–5) for the induction of colitis (Figure 1; Table 1). As in the previous study, the animals were euthanized after a 2-day recovery period following DSS exposure (experimental day 7) for the assessment of colitis and the obtention of samples (see below). Throughout the study, their individual body weight, general state, and the presence of clinical signs associated with the development of colitis were assessed on a daily basis. Mechanical sensitivity (von Frey test) was assessed at the beginning of the studies (experimental day −1, as a baseline measure of sensitivity), during DSS exposure (experimental day 3), and at the peak of inflammation (immediately before euthanasia, experimental day 7) (Figure 1).

### 2.6. Sample Collection

Immediately after the last von Frey test (experimental day 7), the mice were deeply anesthetized with isoflurane (Isoflo; Esteve, Barcelona, Spain) and euthanatized by exsanguination through intracardiac puncture, followed by cervical dislocation. Thereafter, a medial laparotomy was performed, the ceco-colonic region was localized, and the cecum and colon were dissected. Two tissue samples from the proximal-middle colon (about 1.5 cm each) were collected. A sample was frozen immediately in liquid nitrogen. A second sample was fixed in 4% paraformaldehyde. After an overnight fixing, the tissues were paraffin-embedded and 5 µm-thick sections were obtained. Lumbosacral (L3-S2) spinal cord samples were also collected and frozen immediately in liquid nitrogen. The frozen samples were stored at −80 °C until analysis. During the necropsy, the liver, adrenal glands, thymus, and spleen were dissected and weighed.

### 2.7. Clinical and Macroscopic Assessment of Inflammation

The clinical assessment of inflammation included daily monitoring of the body weight, appearance of feces, and general health condition [23]. A score (0–8) was assigned to the health condition (including hunched posture, piloerection, fecal consistency and anal inflammation), where 0 indicates normal activity/fur/fecal content/no anal inflammation, 1 indicates abnormal gait/bristly fur/wet anus/loose fecal content, and 2 indicates prostrated animal/dirty fur/watery or bloody rest on anus/watery diarrhea. During the necropsy, the macroscopic appearance of the colon was scored (macroscopic inflammatory score, 0–15) according to procedures established elsewhere [23]. In brief, the consistency of the fecal contents (score 0–3), the presence of visible fecal blood (score 0–3), the evidence and extent of edema (0–3), the wall thickness (0–3), tissue stiffness (0–2), and presence of ulcerations (0–1) were assessed.

### 2.8. Histological Studies

For the histological examinations, hematoxylin–eosin-stained sections from the colon were obtained following standard procedures. A histopathological score (ranging from 0: normal, to 12: maximal alterations) was assigned to each animal [19,33]. The parameters scored included: epithelial structure (0: normal; 1: mild alterations of the villi; 2: local villi destruction and/or fusion; 3: generalized villi destruction and/or fusion), structure of the crypts (0: normal; 1: mild alterations of the crypts; 2: local destruction of the crypts; 3: generalized destruction of the crypts), presence of edema (0: normal; 1: mild local edema in submucosa and/or lamina propria; 2: moderate diffuse edema in submucosa and/or lamina propria; 3: severe generalized edema in submucosa and/or lamina propria), and presence of inflammatory infiltrate (0: normal; 1: mild localized infiltrate; 2: mild generalized infiltrate; 3: severe generalized infiltrate). Scoring was performed on coded slides by two independent researchers, and the mean value of the two scores was taken as the final score per animal.

### 2.9. Gene Expression: Quantitative Reverse Transcription-PCR

The total RNA was extracted from frozen tissue of the colon and spinal cord samples using TRI reagent with a Ribopure Kit (Ambion/Applied Biosystems, Foster City, CA, USA). Later, a two-step quantitative real-time PCR (RT-qPCR) was performed. The RNA samples were converted into cDNA using a High-Capacity cDNA Reverse Transcription Kit (Applied Biosystems). The PCR reaction mixture was incubated on the Bio-Rad CFX384 Touch Real-Time PCR Detection System (Bio-Rad). All samples were assayed in triplicate. The cycle thresholds for each sample were obtained, and the data were analyzed using the comparative Ct method (2^−ΔΔCt^) with the WT control (for the analysis of the samples originated in the first experiment, including the σ_1_R KO mice) or the vehicle group (for the analysis of the samples generated in the second experiment pharmacological treatments in WT mice) serving as the calibrator [34]. TaqMan^®^ gene expression assays (hydrolysis probes, Applied Biosystems) for interferon γ (INF-γ) (Mm01168134_m1), interleukin 1β (IL-1β) (Mm00434228_m1), interleukin 6 (IL-6) (Mm00446190_m1), interleukin 10 (IL-10) (Mm00439614_m1), interleukin 12 (IL-12p40) (Mm00434174_m1), cannabinoid receptors 1 (CB1) (Mm01212171_s1) and 2 (CB2) (Mm00438286_m1), µ-opioid receptor (MOR) (Mm01188089_m1), tryptophan hydroxylase 1 (TPH1) (Mm00493794_m1), transient receptor potential vanilloid 1 (TRPV1) (Mm01246302_m1), nerve growth factor (NGF) (Mm00443039_m1), metabotropic glutamate receptor 2 (GluR2) (Mm01235831_m1), tachykinin receptor 1 (NK1r) (Mm00436892_m1), neuronal nitric oxide synthase 1 (nos 1, nNOS) (Mm01208059_m1), glutamate receptor ionotropic, NMDA 2B (Grin2b, NR2B) (Mm00433820_m1), and σ1 receptor (σ_1_R) (Mm00448086_m1) were used. β-2-microglobulin (Mm00437762_m1) was used as an endogenous reference gene.

### 2.10. Protein Expression: Western Blot

Lumbosacral spinal cord samples were homogenized by sonication in radioimmunoprecipitation assay (RIPA) buffer, and the supernatant was obtained. Equal amounts of protein (30 µg) were fractionated by 10% (*w*/*v*) SDS–PAGE and transferred onto a polyvinylidene difluoride membrane, blocked with 5% non-fat dry milk in Tris-Tween 20-buffered Saline (T-TBS) for 1 h. The membranes were then incubated in 1% non-fat dry milk in T-TBS overnight at 4 °C with primary antibodies against the protein targets of interest (Table 2). Mouse or rabbit anti-GAPDH antibody or anti-β-tubulin antibody was used as a loading control depending on the protein of interest, and the origin of the primary antibody used. After washing with T-TBS, the blots were incubated for 1 h with secondary peroxidase-conjugated antibodies (see Table 2). Immunoreactive bands were detected by a peroxidase reaction using an enhanced chemiluminescence method (WesternSure^®^ PREMIUM Chemiluminescent Substrate, Li-cor) and the CDiGit ^®^ Blot Scanner (LI-COR). Quantification of the Western blots was carried out with Image Studio™ Lite Software, version 5.2.5.

### 2.11. Statistical Analysis

The data are expressed as the mean ± SEM. A robust analysis (one iteration) was used to obtain the mean ± SEM for the RT-qPCR data. The data were analyzed by one-, two-, or three-way ANOVA, as appropriate, followed, when necessary, by a Bonferroni’s multiple comparisons test. The data were considered statistically significant when *p* < 0.05. Statistical analyses were performed using GraphPad Prism 9 (GraphPad Software, La Jolla, CA, USA) or SPSS program (version 17 for Windows, IBM, Madrid, Spain).

## 3. Results

### 3.1. σ_1_R KO CD1 Mice Develop an Attenuated Acute Colitis

**Figure 2:**
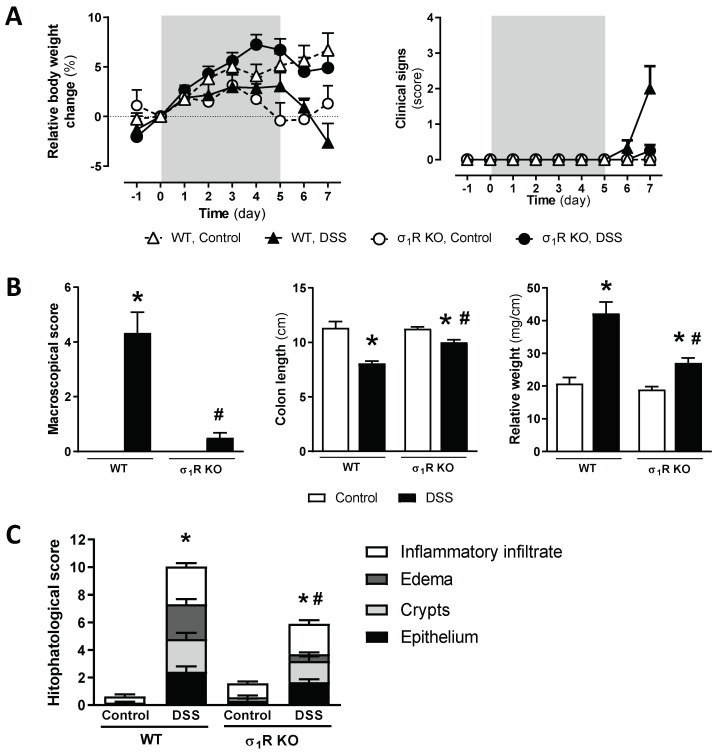
Assessment of DSS-induced acute colitis in WT and σ_1_R KO CD1 mice. (**A**) Changes in relative body weight (% change from day 0, taken as 100%) and clinical signs. The DSS-treatment period is indicated by the grey area. (**B**) Assessment of inflammation-related parameters (macroscopic scores, colonic length, and colon relative weight) as determined during necropsy. (**C**) Histopathological scores of the colon for the different experimental groups. Data are mean ± SEM of 6–8 animals per group. *: *p* < 0.05 vs. respective control group; #: *p* < 0.05 vs. WT mice exposed to DSS.

WT CD1 mice exposed to 3% DSS for 5 consecutive days showed clinical signs consistent with the development of acute colitis, including body weight loss, piloerection, loose feces/watery diarrhea, and the presence of fecal blood. These changes were particularly evident at experimental days 6 and 7. Clinical signs and body weight loss were attenuated in the σ_1_R KO animals exposed to DSS (Figure 2A). A three-way ANOVA revealed significant effects of time (*p* < 0.05), genotype (*p* < 0.05), and DSS exposure (*p* < 0.05) and significant effect interactions among the three factors (*p* < 0.05), thus indicating a different response to DSS over time in both genotypes. Water intake was similar across the experimental groups. In the current experimental conditions, no mortality was observed associated with DSS exposure. 

During the necropsy, macroscopic signs of inflammation, together with a shortening and increased relative weight of the colon, was observed in WT mice receiving DSS (all *p* < 0.05 vs. non-colitic animals; Figure 2B). These inflammation-related parameters were significantly attenuated in the σ_1_R KO mice (*p* < 0.05 vs. DSS-exposed WT mice for the three parameters assessed; Figure 2B). 

Microscopic analysis of the colonic tissue samples showed a normal histological structure in the control animals. Significant histopathological alterations were observed in the WT mice exposed to DSS, reaching a total score of 10.04 ± 1.44 (*p* < 0.05 vs. non-colitic WT mice: 0.63 ± 0.21; Figure 2C and Figure S1). In the healthy σ_1_R KO mice, an essentially normal histological structure was observed. Significant histopathological alterations were observed in the σ_1_R KO mice exposed to DSS (histopathological score; 5.91 ± 0.92; *p* < 0.05 vs. healthy σ_1_R KO mice: 1.57 ± 0.34), although a significant attenuation was observed vs. the WT animals exposed to DSS (*p* < 0.05, Figure 2C and Figure S1). This attenuation was particularly evident, as it relates to the presence of submucosal edema (σ_1_R KO mice: 0.5 ± 0.16; *p* < 0.05 vs. WT mice: 2.54 ± 0.37). 

**Figure 3:**
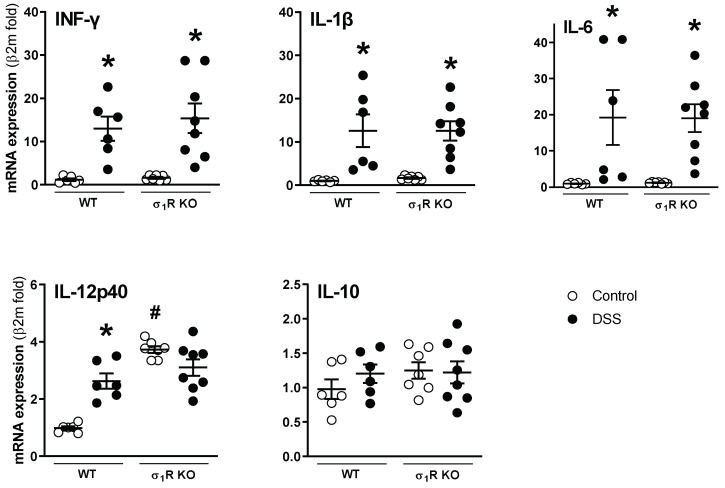
Colonic expression of pro- (interferon-γ (INF-γ), IL-1β, IL-6, and IL-12p40) and anti-inflammatory cytokines (IL-10) in WT and σ_1_R KO CD1 mice. Each point represents an individual animal; the horizontal bar with errors represents the mean ± SEM. *: *p* < 0.05 vs. respective control group; #: *p* < 0.05 vs. control WT mice.

The mRNA of all the analyzed cytokines was detectable and quantifiable by RT-qPCR in the colonic tissues analyzed. The basal expression was similar in the WT and σ_1_R KO mice, except for the pro-inflammatory cytokine IL-12p40, which was upregulated by 4-fold in the σ_1_R KO animals (*p* < 0.05 vs. WT animals; Figure 3). Regardless of the genotype considered, the expression of the pro-inflammatory cytokines INF-γ, IL-1β, and IL-6 was similarly upregulated in the DSS-treated mice (*p* < 0.05 vs. control WT or σ_1_R KO mice; Figure 3). The pro-inflammatory cytokine IL-12p40 was upregulated only in the colitic WT mice (*p* < 0.05 vs. control WT mice). No changes were observed in the expression of the anti-inflammatory cytokine IL-10, regardless of the genotype considered (Figure 3).

### 3.2. σ_1_R KO Mice Do Not Develop Acute Colitis-Associated Hypersensitivity

**Figure 4:**
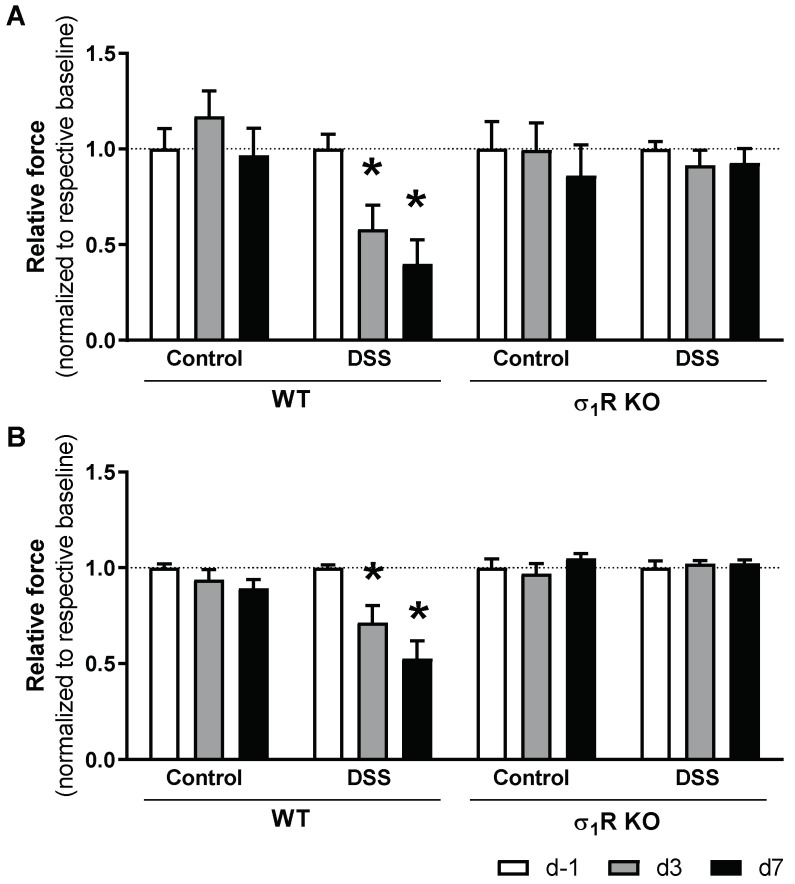
Time-course changes of sensitivity thresholds to mechanical probing of the abdominal wall and hind paw during DSS-induced colitis in WT and σ_1_R KO CD1 mice. The data represent abdominal withdrawal (**A**) and paw withdrawal thresholds (**B**) in WT and σ_1_R KO animals (normalized for measurements at experimental day −1 (d −1) in each experimental group). Data are mean ± SEM of 6 animals per group. *: *p* < 0.05 vs. d −1 of respective group (basal response) and the same time-point in the corresponding control group.

The WT and σ_1_R KO mice showed similar baseline abdominal (WT: 1.48 ± 0.02 g; σ_1_R KO: 1.43 ± 0.04 g; *p* > 0.05) and paw withdrawal thresholds (WT: 1.19 ± 0.08 g; σ_1_R KO: 1.21 ± 0.09 g; *p* > 0.05), as assessed with the von Frey test immediately before colitis induction (experimental day −1). During the development of acute colitis, the WT mice developed referred hyperalgesia, manifested as a time-related, progressive reduction in the paw and abdominal withdrawal thresholds (both *p* < 0.05 vs. respective sensitivity thresholds in basal conditions). In the control animals not exposed to DSS, paw and abdominal withdrawal thresholds remained stable over time (Figure 4).

Similar to that observed in the WT animals, the σ_1_R KO mice not exposed to DSS showed a stable pain sensitivity throughout the experimental time (revealed by the absence of changes in the withdrawal thresholds). Moreover, no changes in pain sensitivity were detected in the σ_1_R KO mice exposed to DSS. In these animals, paw and abdominal withdrawal thresholds remained stable over time and were similar to the basal values (determined in the same animals before colitis induction) or to the withdrawal thresholds determined in the animals without colitis (Figure 4).

**Figure 5:**
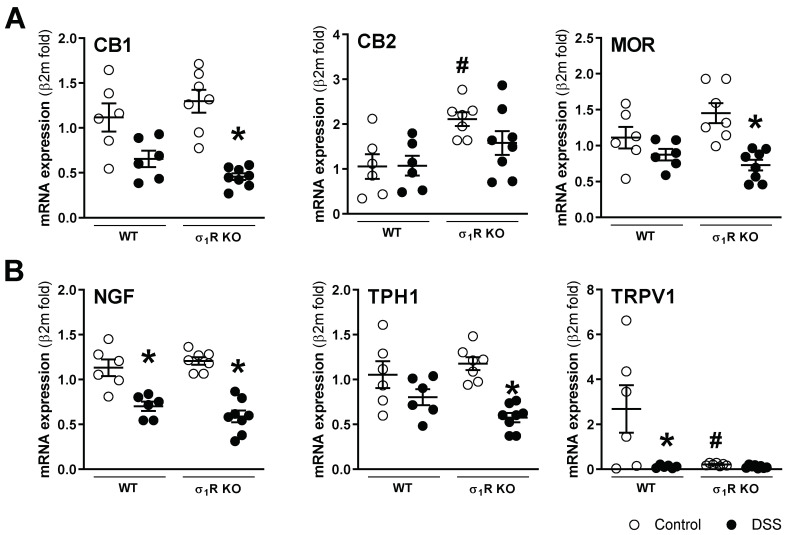
Colonic gene expression of sensory-related markers with anti-nociceptive ((**A**) CB1, CB2, and MOR) and pro-nociceptive activity ((**B**) NGF, TPH1, and TRPV1) in the different experimental groups. Each point represents an individual animal; the horizontal bar with errors represents the mean ± SEM. *: *p* < 0.05 vs. respective non DSS-exposed control group; #: *p* < 0.05 vs. WT control.

mRNA for the sensory-related markers assessed was detected in all colonic samples, with the exception of σ_1_Rs, which, as expected, was not detected in the KO mice. A differential gene expression regulation was observed during colitis. The basal expression of the anti-nociceptive (CB1 and MOR) and pro-nociceptive markers (NGF, TPH1) was similar in the WT and σ_1_R KO animals. However, a significant upregulation of the CB2 receptors (anti-nociceptive) and a significant downregulation of TRPV1 (pro-nociceptive) were detected in the σ_1_R KO control mice (both *p* < 0.05 vs. expression levels in WT animals; Figure 5). During colitis, similar modulation in the expression of sensory-related markers was observed in both genotypes, where the NGF mRNA levels were downregulated by colitis in both genotypes (*p* < 0.05 vs. healthy WT and σ_1_R KO mice, respectively; Figure 5). The anti-nociceptive markers showed a general tendency towards downregulation, although statistical significance was only achieved for CB1 and MOR in the σ_1_R KO animals. Similarly, the pro-nociceptive markers showed, in all cases, a downregulatory trend, achieving a significant effect for TPH1 expression in the WT mice (*p* < 0.05 vs. healthy WT mice, Figure 5). In the WT mice, colitis did not affect the colonic gene expression of σ_1_R (Supplementary Figure S2).

**Figure 6:**
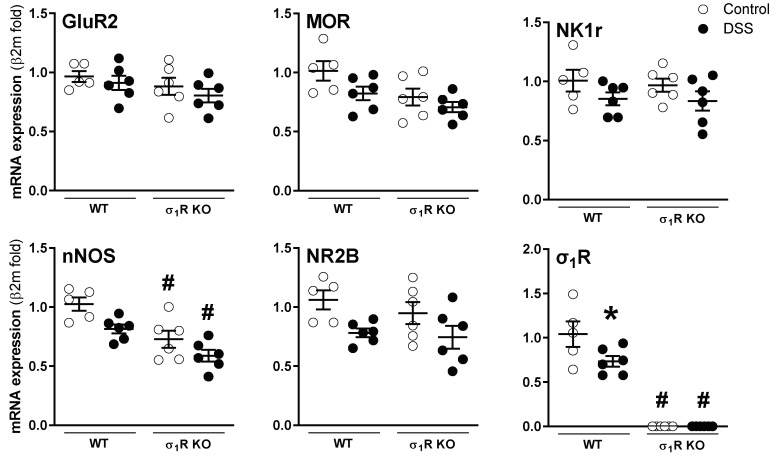
Gene expression of sensory pathway-related markers in the lumbosacral spinal cord of WT and σ_1_R KO CD1 mice with or without colitis. Each point represents an individual animal; the horizontal bar with errors represents the mean ± SEM. *: *p* < 0.05 vs. respective control group; #: *p* < 0.05 vs. respective WT mice group.

At the spinal level, mRNA of the sensory pathway-related markers assessed was detected in all samples. Overall, neither genotype- nor inflammation-related changes were detected, with the exception of nNOS and σ_1_Rs. As it relates to nNOS, a constitutive downregulation was detected in the σ_1_R KO mice (*p* < 0.05 vs. WT mice; Figure 6). As expected, the expression of σ_1_Rs was not detected in the spinal cord of the KO mice. Moreover, during colitis, the σ_1_R expression was downregulated by 30% in the WT mice (*p* < 0.05 vs. healthy WT mice; Figure 6). The presence of proteins implicated in the sensitization mechanisms, namely pERK, pCaMKII, pp38, and GFAP, was detected in the lumbosacral spinal cord in all samples analyzed, although, with relatively large variability in some cases. Overall, no consistent genotype- or inflammation-related changes were detected (Supplementary Figure S3).

### 3.3. Antagonism of σ_1_Rs with BD1063 or E-52862 Attenuates DSS-Induced Colitis

**Figure 7:**
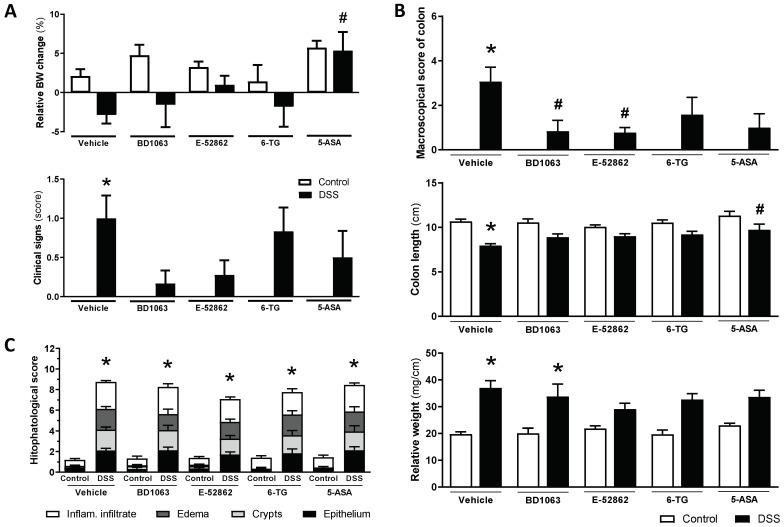
Assessment of DSS-induced acute colitis in WT CD1 mice and effects of pharmacological treatments. (**A**) Changes in relative body weight (% change from day 0, taken as 100%) and clinical signs associated with exposure to DSS and pharmacological treatments (σ_1_R antagonists, BD1063 or E-52862, 6-TG or 5-ASA) at experimental day 7. (**B**) Assessment of colonic inflammation (inflammatory score, colon length, and colon relative weight) at the time of necropsy in the different experimental groups. (**C**) Histopathological scores in the different experimental groups. Data are mean ± SEM of 6–15 animals per group. *: *p* < 0.05 vs. respective control group; #: *p* < 0.05 vs. DSS-vehicle-treated group.

The vehicle-treated WT mice receiving DSS showed weight loss and clinical signs consistent with the induction of a colitic state. Treatment with BD1063 or E-52862 resulted in a similar attenuation of body weight loss and clinical signs, although statistical significance was not achieved, probably because of the relatively large variability observed in some cases (Figure 7A).

During the necropsy, colonic alterations consistent with the development of colitis, increased inflammatory scores, colon shortening, and an increase in the relative weight were observed in the vehicle-treated mice exposed to DSS (Figure 7B). The antagonism of σ_1_Rs attenuated some of these parameters, but different effects were observed for BD1063 (reduction of inflammatory scores; *p* < 0.05 vs. control) and E-52862 (reduction of inflammatory scores and relative weight; both *p* < 0.05 vs. control). However, histopathological alterations were not affected by either BD1063 or E-52862. Indeed, the animals receiving BD1063 or E-52862 showed essentially the same structural alterations and histopathological scores as those of the vehicle-treated animals also exposed to DSS (Figure 7C).

The reference compounds, 6-TG and 5-ASA, attenuated the macroscopical scores of inflammation (although statistical significance was not achieved) and the increase in the relative colonic weight (*p* > 0.5 vs. relative weight in non-colitic animals), without improving the histopathological alterations (Figure 7).

**Figure 8:**
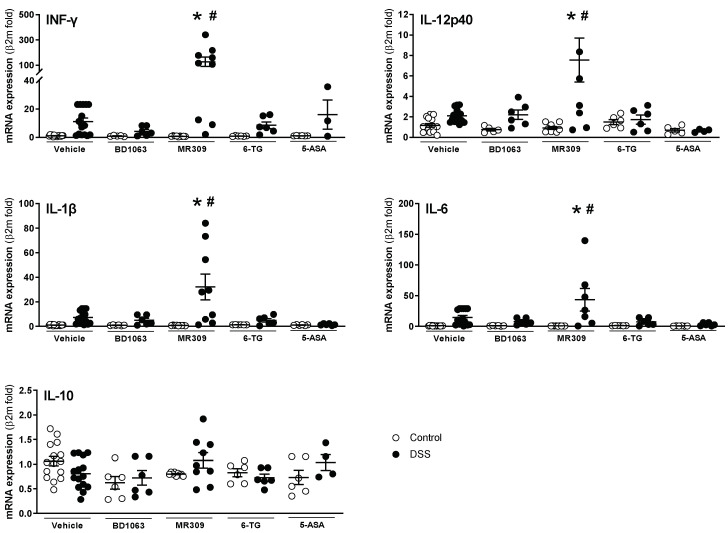
Colonic expression of pro-(interferon-γ (INFγ), IL-1β, IL-6, and IL-12p40) and anti-inflammatory cytokines (IL-10) in animals with DSS-induced colitis treated with the selective σ_1_R antagonists (BD1063 or E-52862), 6-TG, or 5-ASA. Each point represents an individual animal; the horizontal bar with errors represents the mean ± SEM. *: *p* < 0.05 vs. respective non DSS-exposed control group; #: *p* < 0.05 vs. vehicle-treated mice receiving DSS.

mRNA expression of all the analyzed cytokines was detectable and quantifiable by RT-qPCR in all samples. In the vehicle-treated animals, DSS exposure led to expression changes equal to those observed in the first study, comparing WT and σ_1_R KO animals. However, due to the variability observed, statistical significance was not achieved (Figure 8). In animals exposed to DSS, BD1063 showed a tendency to normalize the expression of the pro-inflammatory cytokines INF-γ, IL-1β, and IL-6, without affecting the expression of IL-12p40. On the contrary, treatment with E-52862 resulted in a significant increase in the expression of all the pro-inflammatory cytokines assessed, although with high inter-individual variability (Figure 8). 6-TG and 5-ASA showed moderate effects, with a tendency to reduce the expression of pro-inflammatory cytokines, with the exception of INF-γ, which, in the 5-ASA-DSS-treated animals, showed an upregulation with high interindividual variability (Figure 8). No changes were observed in the expression of the anti-inflammatory cytokine IL-10, regardless of the treatment considered.

### 3.4. Antagonism of σ_1_Rs with BD1063 or E-52862 Attenuated Colitis-Associated Hypersensitivity

**Figure 9:**
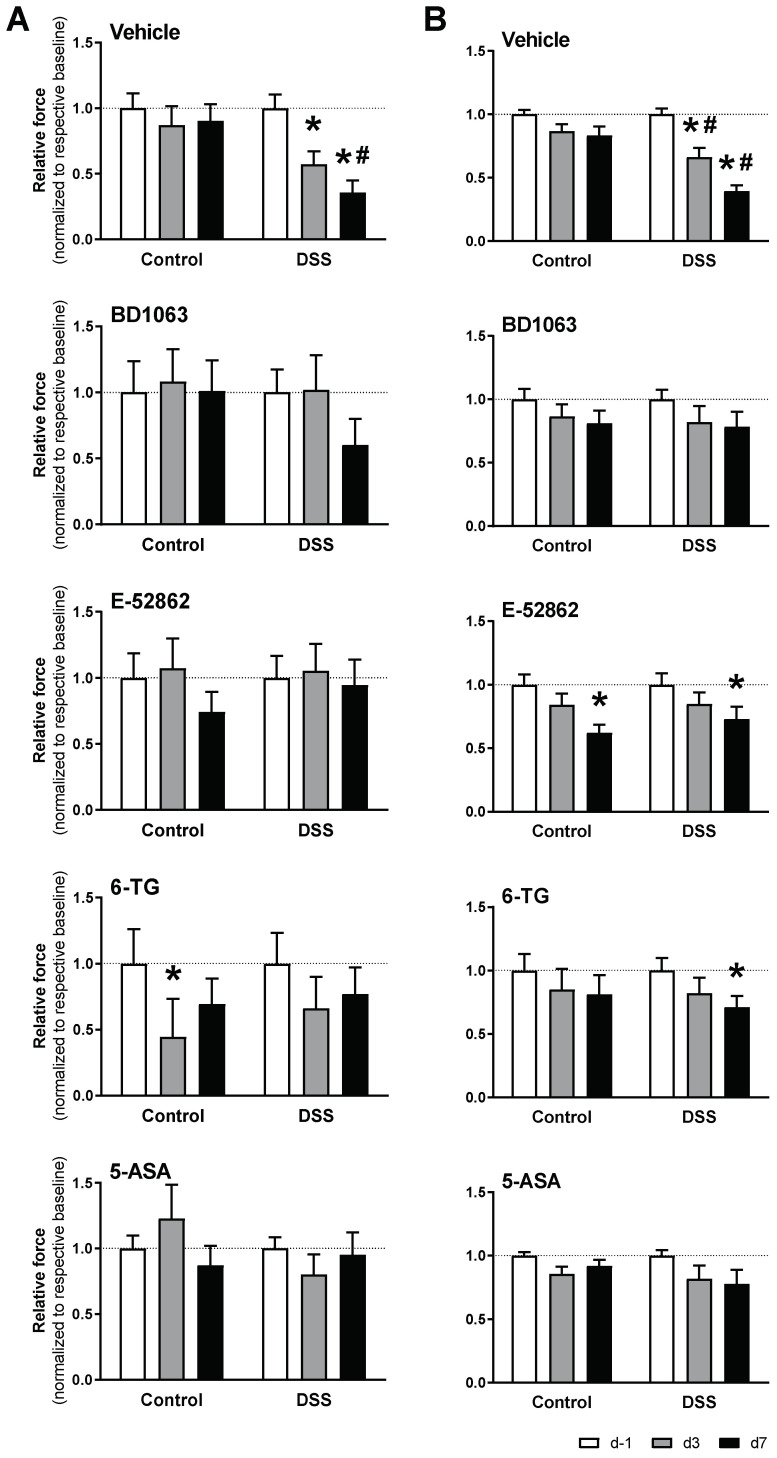
Effects of σ_1_R antagonists (BD1063 or E-52862), 6-TG, and 5-ASA on acute colitis-associated hypersensitivity. The data represent abdominal (**A**) and paw withdrawal thresholds (**B**) (normalized for measurements at experimental day −1 (d −1) in each experimental group). Data are mean ± SEM of n = 6–15 animals per group. *: *p* < 0.05 vs. d −1 of respective group (basal response); #: *p* < 0.05 vs. respective control group.

As expected, the vehicle-treated mice exposed to DSS developed referred hyperalgesia, manifested as a time-related, progressive reduction in the withdrawal thresholds determined in the paw and the abdominal wall (both *p* < 0.05 vs. respective sensitivity thresholds in non-inflamed animals). In the control animals not exposed to DSS, the paw and abdominal withdrawal thresholds remained stable over time (Figure 9).

Treatment with BD1063 completely prevented the development of hypersensitivity, as assessed either in the paw or in the abdominal wall. On the other hand, treatment with E-52862, while completely preventing the development of hyperalgesia as assessed at the abdominal wall, only partially prevented the sensitivity changes observed at the paw (Figure 9).

Similar inhibitory effects were observed for 6-TG and 5-ASA. Treatment with 5-ASA completely prevented colitis-associated hypersensitivity, while in 6-TG-treated animals, paw hyperalgesia was detected only at experimental day 7 (Figure 9).

**Figure 10:**
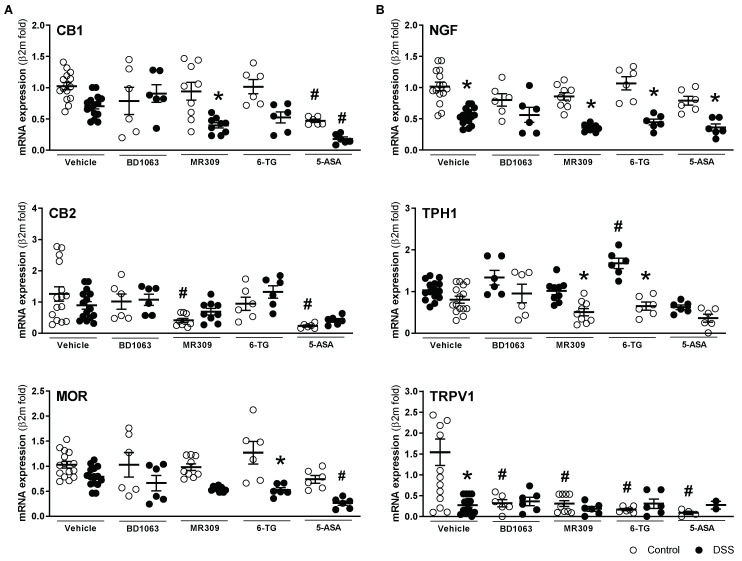
Colonic gene expression of sensory-related markers with antinociceptive ((**A**) CB1, CB2 and MOR), and pro-nociceptive activity ((**B**) NGF, TPH1, and TRPV1) in the different experimental groups. Each point represents an individual animal; the horizontal bar with errors represents the mean ± SEM. *: *p* < 0.05 vs. respective control group; #: *p* < 0.05 vs. control-vehicle-treated group.

mRNA expression of all sensory-related markers assessed was detected in all colonic samples. Overall, the gene expression changes detected in the vehicle-treated mice exposed to DSS were similar to those reported in the first experiment, with a tendency for a general downregulation during colitis, although in this case, statistical significance was only reached for NGF and TRPV1 (Figure 10). Treatment with BD1063 had no significant effects on gene expression (healthy or colitic animals), with the exception of TRPV1, which was significantly downregulated in healthy animals (Figure 10). Treatment with E-52862 resulted in a downregulation of the expression of CB2 and TRPV1 in healthy animals. During colitis, the E-52862-treated animals presented an overall downregulation in the expression of sensory markers, similar to that observed in the vehicle-treated animals, but in this case, statistical significance was achieved for the expression of CB1, NGF, and TPH1 (all *p* < 0.05 vs. E-52862-treated animals not exposed to DSS; Figure 10). 6-TG, per se, had minor effects on gene expression, with the more relevant effect being the downregulation of TRPV1 expression (*p* < 0.05 vs. vehicle-treated healthy mice; Figure 10). In the animals with colitis receiving 6-TG, changes in the gene expression were comparable to those observed in the vehicle-treated colitic mice. In the healthy animals, 5-ASA led to the downregulation of the CB1 and CB2 receptors in addition to that of TRPV1 (all *p* < 0.05 vs. vehicle-treated healthy mice). During colitis, a similar general downregulation of sensory markers described in the vehicle-treated animals was detected in the 5-ASA-treated animals (Figure 10). Regardless of the treatment considered or the presence or not of inflammation, no changes were detected for the colonic expression of σ_1_Rs (Supplementary Figure S4).

## 4. Discussion

Previous studies have demonstrated the involvement of σRs in different pain models [19,35,36]. This study expands this knowledge by assessing the implication of σ_1_Rs in the development of hypersensitivity associated with intestinal inflammation. The present results confirm that σ_1_Rs are implicated in the development of acute inflammation within the GI tract and the associated changes in referred sensitivity at the paw (somatic) and abdominal wall (likely implicating somatic and visceral perception). Two different approaches were used to characterize the role of σ_1_Rs; the genetic, constitutive absence of the receptor using KO mice for the receptor, and the pharmacological blockade with the selective σ_1_R antagonists BD1063 and E-52862.

Intestinal inflammation associated with sensorial alterations characterized by changes in somatic and visceral sensitivity (somatic and visceral hyperalgesia) are common findings in several GI pathologies, mainly in inflammatory bowel disease and functional GI disorders (with irritable bowel syndrome as the main representative) [1,2,3]. Several animal models have been designed to mimic inflammatory conditions of the gut. Among them, DSS-induced colitis is a well validated and accepted model of intestinal inflammation. Upon DSS exposure, mice develop a state of acute colitis reminiscent of the flares observed in humans with inflammatory bowel disease. The responses observed are strain-related [22,23]. In CD1 mice, this response occurs 7–9 days after starting DSS exposure, with the presence of a flare of acute colitis, and resolves by day 14 [31,32]. Consistent with these observations, in the present experimental conditions, CD1 mice receiving 3% DSS developed an acute state of colitis characterized by the presence of clinical signs, body weight loss, histopathological alterations, and the upregulation of pro-inflammatory cytokines. Overall, these alterations agree with those described in comparable experimental conditions [31,32] and are consistent with an acute inflammatory response. 

Overall, the results obtained in the present report in σ_1_R KO CD1 mice coincide with those obtained previously in the C57BL/6 strain [19], with only minor, likely strain-related, differences. Supporting the involvement of the σ_1_R in the development of intestinal inflammation, the attenuation of clinical signs and changes in body weight were observed in the σ_1_R KO animals exposed to DSS. The apparent resistance to inflammation in the σ_1_R KO mice is further supported by the macroscopic assessment of the colon at the time of necropsy, showing an attenuation of the inflammation-related parameters vs. the WT animals. This was further confirmed by the attenuation of colonic histopathological alterations. The histopathological improvement was associated mainly with a reduction in the submucosal edema, thus agreeing with our previous results in C57BL/6 mice with DSS-induced colitis [19] and with the reduction of subepithelial edema in σ_1_R KO CD1 mice in a model of cyclophosphamide-induce cystitis [18] or the reduction in paw edema, elicited by the intraplantar injection of carrageenan during the pharmacological blockade of σ_1_Rs [37,38]. Although the mechanisms mediating these effects have not been fully elucidated, they might implicate a NOS-dependent modulation of vascular permeability and extravasation [37,38]. Further supporting a role for σ_1_Rs in intestinal inflammation, the pharmacological blockade of the receptor in the WT mice partially reproduced the responses observed in the σ_1_R KO mice. Both BD1063 and E-52862 attenuated clinical signs of colitis and improved colonic macroscopic signs of inflammation. However, at the histopathological level, no signs of improvement were observed, particularly when considering submucosal edema. This agrees with previous studies showing that the pharmacological blockade with the same antagonists used here failed to reduce paw edema in inflammatory pain models [11,14,15]. Overall, this might represent limits of the pharmacological blockade of σ_1_Rs vs. their constitutive absence in KO animals. 6-TG and 5-ASA modulated inflammation in a similar manner, with an improvement in the clinical and macroscopic signs of inflammation, but without amelioration of the histopathological scores.

The clinical attenuation of colitis observed in the σ_1_R KO mice or during the treatment with selective σ_1_R antagonists did not correlate with the gene expression of pro-inflammatory cytokines, which were upregulated in similar proportions to those detected in the control conditions. Overall, the colonic expression of pro- (INFγ, IL-1β, and LI-6) and anti-inflammatory cytokines (IL-10) was similar in the non-inflamed WT and σ_1_R KO mice. However, IL-12p40 was constitutively upregulated in the KO mice, thus suggesting that these animals might be prone to developing immune-mediated responses. Indeed, previous evidence suggests an immunomodulatory role for σ_1_Rs, pointing towards potential anti-inflammatory activity [39]. Accordingly, a lack of functional σ_1_Rs, as in the σ_1_R KO mice, should translate into enhanced inflammatory responses. However, and according to what was observed experimentally, compensatory mechanisms in these animals might lead to a state in which inflammatory responses are constitutively attenuated [40]. Moreover, in the WT animals exposed to DSS, treatment with E-52862 led to an unexpected significant upregulation of all the pro-inflammatory cytokines assessed, while no expression changes were observed in the healthy animals. The underlying mechanisms mediating these effects remain unknown. In any case, this reinforces the potential immunomodulatory role of σ_1_Rs during inflammation and highlights differences in the pharmacological profiles of E-52862 and BD1063. However, the E-52862-mediated upregulation of pro-inflammatory cytokines was not associated with a worsening of the inflammatory process. As expected, 6-TG and 5-ASA showed a similar modulatory action on cytokine expression, with an overall tendency towards normalization, in accordance with their anti-inflammatory activity.

Baseline mechanical sensitivity, as assessed in the hind limbs or the abdominal wall, was similar in the WT and σ_1_R KO mice, in agreement with previous data indicating that naive σ_1_R KO mice perceive mechanical and thermal stimuli normally [10,11,19]. Moreover, the pharmacological blockade of σ_1_Rs in healthy animals did not affect, per se, mechanical sensitivity, with the exception of a punctual referred hyperalgesia observed in the E-52862-treated mice when assessing paw sensitivity. Overall, this agrees with studies with σ antagonists showing that σ_1_R ligands have no effects by themselves, but are able to modulate signaling pathways under pathological conditions [10,12,13]. The reduction in the referred sensitivity thresholds observed in the E-52862-treated animals, either with or without acute colitis, might reflect a compound-related hyperalgesic effect. However, to the best of our knowledge, similar effects have not been described before for E-52862. This finding further emphasizes differences in the pharmacological profile of E-52862 and BD1063.

Intestinal hypersensitivity has been associated with the peripheral sensitization of primary colonic sensory afferents and referred hyperalgesia in several body regions, including the abdominal wall, hind paws, and tail [1,4,5,41]. In the DSS-induced colitis model, persistent harmful stimulation of visceral nociceptors through inflammatory mediators would be responsible for inducing acute visceral and somatic referred hyperalgesia [42]. Accordingly, the WT control mice exposed to DSS developed referred hypersensitivity, as determined by reductions in the pain thresholds to the mechanical probing of the hind paws and the abdominal wall (likely reflecting mixed somatic and visceral (intestinal) pain responses). In contrast, no changes in the mechanical sensitivity were observed in the σ_1_R KO mice. Likewise, the selective σ_1_R antagonists BD1063 and E-52862 effectively reduced inflammation-induced hypersensitivity, leading to, essentially, the same responses observed in control conditions or in the σ_1_R KO mice. This agrees with the reported analgesic effects of BD1063 on models of intracolonic capsaicin- and cystitis-induced visceral pain [17,18]. Altogether, these results confirm a role of σ_1_Rs in the development of visceral inflammation-associated hypersensitivity, as previously reported from other models of inflammatory pain [11,14,15,37,38], and they support the view that the antagonism of σ_1_Rs might represent a feasible approach for the treatment of pain arising from the GI tract. 

To gain insight into the mechanisms implicated on nociceptive responses, we assessed the expression of different sensory-related markers, both at the level of the colon and within the lumbosacral spinal cord. Except for CB2 receptors and TRPV1, the basal expression of sensory-related markers was similar in the WT and σ_1_R KO mice. The overexpression of CB2 receptors in the σ_1_R KO mice might be related to the potential immunomodulatory effects described for σ_1_Rs [39], and also observed here through the upregulation of IL-12p40, since CB2 receptors are largely associated with immune cells [43]. On the other hand, the downregulation of TRPV1 suggests a direct interaction between σ_1_Rs and the activity of sensory afferents, since within the colon, TRPV1 is primarily expressed on primary sensory afferents mediating pro-algesic responses [44,45]. Interestingly, treatment with BD1063 or E-52862 also resulted in a significant downregulation of the colonic expression of TRPV1. These data are in line with observations indicating that the antagonism of σ_1_Rs is able to decrease nociceptive responses through the negative regulation of TRPV1 protein expression in plasma and in the membrane of sensory neurons [46,47]. Therefore, an altered TRPV1-σ_1_R interaction might contribute to the underlying mechanisms explaining the lack of hypersensitivity in the absence of functional σ_1_Rs. All together, these changes suggest a basal analgesic state in σ_1_R KO animals. However, as described above, the σ_1_R KO mice exhibited normal basal mechanical sensitivity [10,11,19], thus suggesting the presence of additional compensatory mechanisms, as discussed above. In any case, these observations further reinforce the implication of σ_1_Rs on pain mechanisms. Further emphasizing a complex relationship among TRPV1-, σ_1_R-, and MOR-modulating nociception, a recent report suggests that the σ_1_ antagonism might increase MOR activity in TRPV1-positive sensory neurons, thus facilitating the analgesic effects of endogenous opioids and reducing hyperalgesia [48]. During colitis, a general downregulation of colonic sensory-related markers, either with analgesic or pro-algesic activity was observed. Again, this was particularly evident for TRPV1. Colonic expression of TRPV1 might be highly variable with experimental model- and species/strain-related variations. Indeed, ulcerative colitis patients show a reduced expression of TRPV1, while during DSS-induced intestinal inflammation, no changes (acute inflammation) or increased expression (chronic inflammation) has been reported [49,50]. Given the pro-nociceptive effects of TRPV1, its downregulation during acute colitis might be interpreted as a compensatory mechanism developed during acute inflammation to avoid abnormal excessive pain. Overall, it is difficult to establish a direct correlation between the gene expression of sensory-related markers and functional outcomes as it relates to pain sensitivity since a trend towards a downregulation was observed for anti- and pro-nociceptive markers. In this situation, the final functional responses will depend upon the balance between anti- and pro-nociceptive mechanisms, as previously suggested [51,52,53].

In addition to peripheral locations, pain sensitization can occur also at a central level. To assess the potential participation of central (spinal) sensitization in the responses observed during acute colitis, we assessed the lumbosacral expression (gene or protein levels) of several markers related to the spinal processing of sensory-related signals and the sensitization process. Overall, with the exception of the expression of σ_1_Rs and nNOS, no differences between the WT and KO or DSS-treated and untreated animals were detected. nNOS expression was constitutively downregulated in the lumbosacral spinal cord of the σ_1_R KO mice. Recent evidence indicates that during peripheral neuropathy, spinal σ_1_R-induced pain hypersensitivity is mediated by nNOS activation [54,55]. Moreover, spinal activation of the σ_1_R by agonists increased nNOS activity and nitric oxide (NO) production, which led to the development of hypersensitivity [38]. Therefore, it is feasible to assume that the constitutive lack of σ_1_Rs might have consequences in the activity and/or expression of components of associated signaling processes involved in pain sensitization, such as nNOS. Alterations of this pathway might be part of the underlying mechanisms explaining the absence of sensitization during acute colitis in σ_1_R KO mice. In this context, it is noteworthy that, in WT animals, colitis induced a σ_1_R downregulation within the lumbosacral spinal cord. This could act as a compensatory mechanism, regulating sensitization pathways in a negative manner, such as the nNOS pathway discussed above, with the objective of avoiding aberrant/excessive sensitization. These observations warrant further studies to dissect these pathways in detail.

Since only changes associated with acute inflammation were assessed in this work, we cannot discard the implication of these mechanisms in a process of inflammation-related long-term vs. early acute sensitization. For instance, in models of inflammatory/neuropathic pain, the modulation of some of these pathways (i.e., ERK phosphorylation) was observed in the spinal cord up to 14 days after the application of the sensitizing stimuli [10,56]. However, acute modulation of the same pathways during inflammation or neuropathic damage has also been described [57,58]. In any case, the present results agree with our previous findings during acute and chronic colitis in C57BL/6 mice, where we were also unable to demonstrate changes in pain-related pathways within the spinal cord, regardless of the presence of hypersensitivity [19]. Therefore, we cannot discard that multiple factors, including experimental model-, species/strain- and time of testing-related, might account for these apparent discrepancies. Alternatively, the implication of other specific mechanisms acting in visceral sensitization cannot be discarded.

In summary, the present results, based on the genetic ablation or the pharmacological blockade of σ_1_Rs in a model of acute intestinal inflammation, show that σ_1_Rs have positive modulatory effects on intestinal inflammation as well as on the development of inflammation-associated pain, likely preventing both somatic and visceral hypersensitivity. These observations, together with previous evidence in similar models, support the pharmacological interest of σ_1_R antagonists for the treatment of intestinal inflammation and inflammation-associated hypersensitivity, with potential for their clinical use in inflammatory and functional GI disorders.

## Figures and Tables

**Table 1:** Summary of experimental groups.

	Genotype	Colitis Induction	Treatment	n
**Study 1**	Wild-type	No (Tap water)	-	6
		Yes (3% DSS)	-	6
	σ_1_R knockout	No (Tap water)	-	8
		Yes (3% DSS)	-	8
**Study 2**	Wild-type	No (Tap water)	Vehicle (5 mL/kg, po, BID)	15
			BD1063 (20 mg/Kg, po, BID)	6
			E-52862 (20 mg/Kg, po, BID)	9
			6-TG (2 mg/Kg, po, SID)	6
			5-ASA (50 mg/Kg, po, BID)	6
		Yes (3% DSS)	Vehicle (5 mL/kg, po, BID)	15
			BD1063 (20 mg/Kg, po, BID)	6
			E-52862 (20 mg/Kg, po, BID)	9
			6-TG (2 mg/Kg, po, SID)	6
			5-ASA (50 mg/Kg, po, BID)	6

Abbreviations: po: oral; BID: twice a day; SID: once a day.

**Table 2:** Details of antibodies used for Western blot.

Type	Reactivity	Host	Dilution	Source
Primary	β-tubulin	Goat polyclonal	1:1000	Santa Cruz Biotech. (#sc-9935)
Primary	CaMKII	Mouse monoclonal	1:2000	Invitrogen (#MA1-048)
Primary	pCaMKII	Mouse monoclonal	1:1000	Invitrogen (#MA1-047)
Primary	GFAP	Mouse monoclonal	1:10,000	Cell Signaling (#3670)
Primary	GAPDH	Mouse monoclonal	1:80,000	Sigma-Aldrich (#G8795)
Primary	GAPDH	Rabbit polyclonal	1:20,000	Sigma-Aldrich (#G9545)
Primary	tERK	Rabbit polyclonal	1:30,000	Sigma-Aldrich (#M5670)
Primary	pERK	Mouse monoclonal	1:1000	Sigma-Aldrich (#M8159)
Primary	p38	Rabbit polyclonal	1:1000	Invitrogen (#AHO1202)
Primary	pp38	Rabbit monoclonal	1:1000	Invitrogen (#MA5-15177)
Secondary	Anti-Mouse IgG	Goat polyclonal	1:2000	Sigma-Aldrich (#A5278)
Secondary	Anti-Rabbit IgG	Goat polyclonal	1:4000	Sigma-Aldrich (#A9169)
Secondary	Anti-Goat IgG	Donkey polyclonal	1:2000	Abcam (#ab97110)

## Data Availability

All the data presented in this study are available upon request from the corresponding author.

## Supplementary Materials

The following supporting information can be downloaded at: https://www.mdpi.com/article/10.3390/biomedicines11102758/s1, Figure S1: Histopathological assessment of the colon. A–D: Representative microphotographs showing hematoxilin and eosin-stained colonic slices from WT vehicle-treated mice (A), DSS-treated WT mice (B), σ_1_R KO vehicle-treated mice (C) and DSS-treated σ_1_R KO mice (D). Notice the submucosal edema observed in DSS-treated WT mice (B), while it was markedly reduced in DSS-treated σ_1_R KO mice (D). Scale bar: 200 µm; Figure S2: Colonic gene expression of σ_1_Rs in WT and σ_1_R KO CD1 mice. No expression was detected in σ_1_R KO mice. Each point represents an individual animal; the horizontal bar with errors represents the mean ± SEM; Figure S3: Relative levels of pERK (A), pCaMKII (B), pp38 (C) and GFAP (D) in the lumbosacral spinal cord of WT and σ_1_R KO CD1 mice with or without colitis. Data are mean ± SEM of 6–8 animals per group; Figure S4: Effects of σ1R antagonists (BD1063 or E-52862), 6-TG and 5-ASA on colonic gene expression of σ_1_Rs. Each point represents an individual animal; the horizontal bar with errors represents the mean ± SEM.
