# Supplementary material for: Genetic and Pharmacological Blockade of Sigma-1 Receptors Attenuates Inflammation-Associated Hypersensitivity during Acute Colitis in CD1 Mice
Source: Biomedicines. 2023 Oct 12;11(10):2758. doi: 10.3390/biomedicines11102758 (PMC10604167; doi:10.3390/biomedicines11102758)

Genetic and pharmacological blockade of Sigma-1 receptors attenuates inflammation-associated hypersensitivity during acute colitis in CD1 mice

Sergio López-Estévez, Mònica Aguilera, Georgia Gris, Beatriz de la Puente, Alicia Carceller and Vicente Martínez

Supplementary Material

Figure S1. Histopathological assessment of the colon. A-D: Representative microphotographs showing hematoxilin and eosin-stained colonic slices from WT vehicle-treated mice (A), DSS-treated WT mice (B), σ1R KO vehicle-treated mice (C) and DSS-treated σ1R KO mice (D). Notice the submucosal edema observed in DSS-treated WT mice (B), while it was markedly reduced in DSS-treated σ1R KO mice (D). Scale bar: 200 μm.

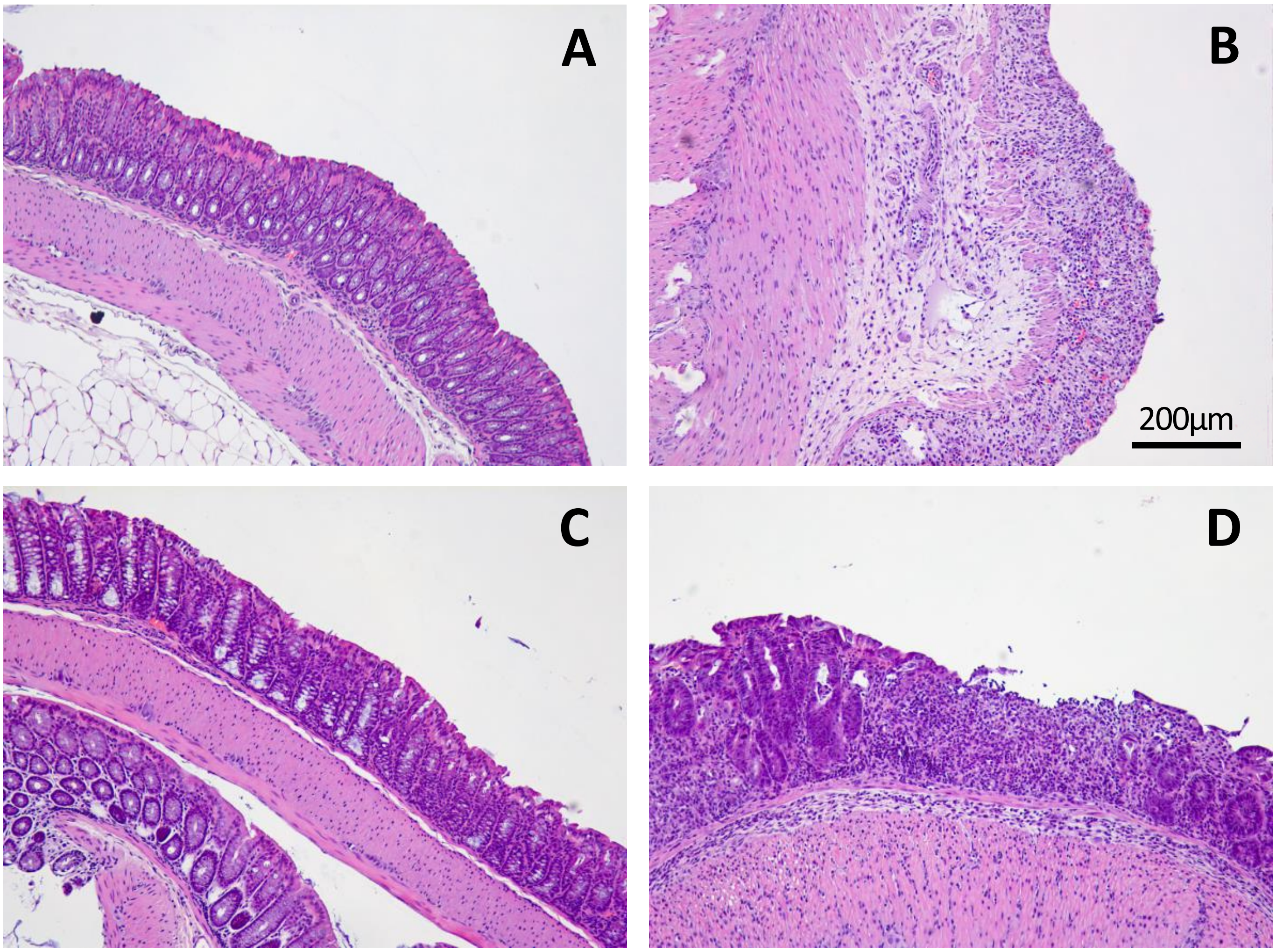

Figure S2. Colonic gene expression of σ1Rs in WT and σ1R KO CD1 mice. No expression was detected in σ1R KO mice. Each point represents an individual animal; the horizontal bar with errors represents the mean±SEM.

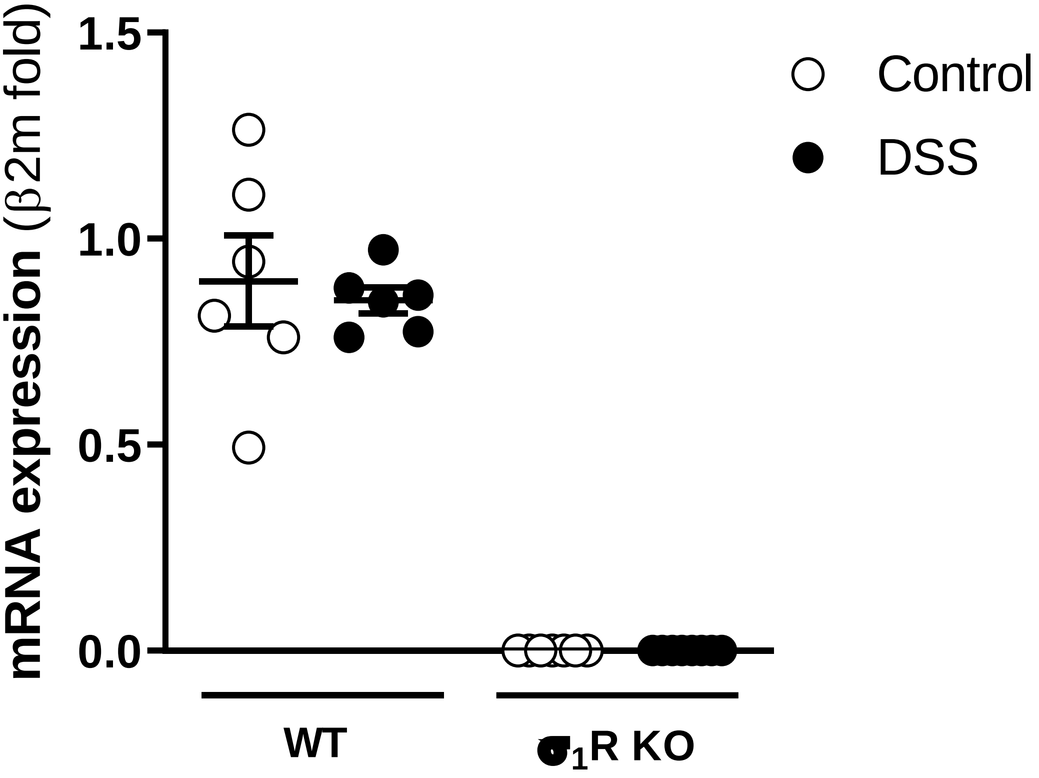

Figure S3. Relative levels of pERK (A), pCaMKII (B), pp38 (C) and GFAP (D) in the lumbosacral spinal cord of WT and σ1R KO CD1 mice with or without colitis. Data are mean±SEM of 6-8 animals per group.

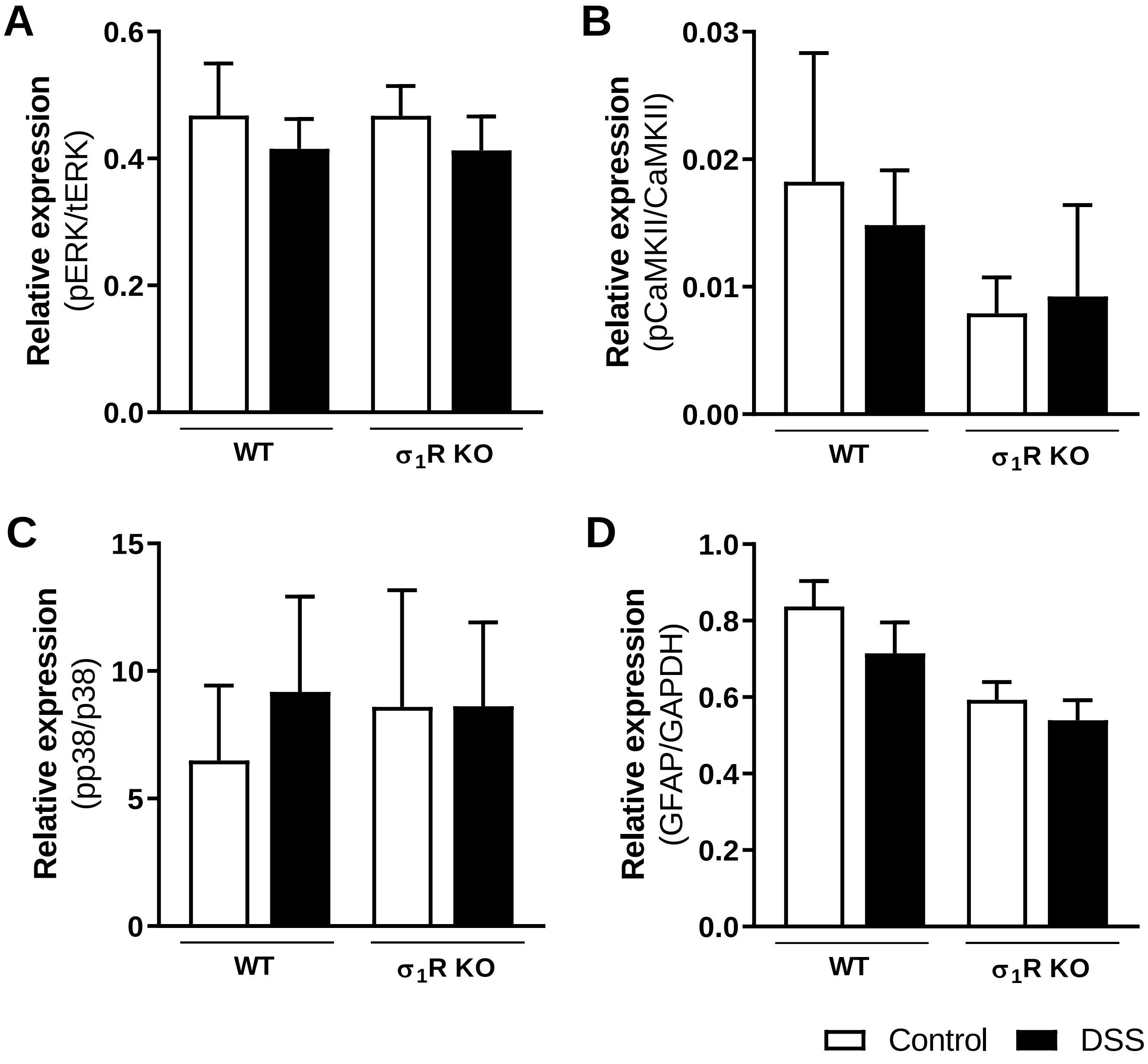

Figure S4. Effects of σ1R antagonists (BD1063 or E-52862), 6-TG and 5-ASA on colonic gene expression of σ1Rs. Each point represents an individual animal; the horizontal bar with errors represents the mean±SEM.

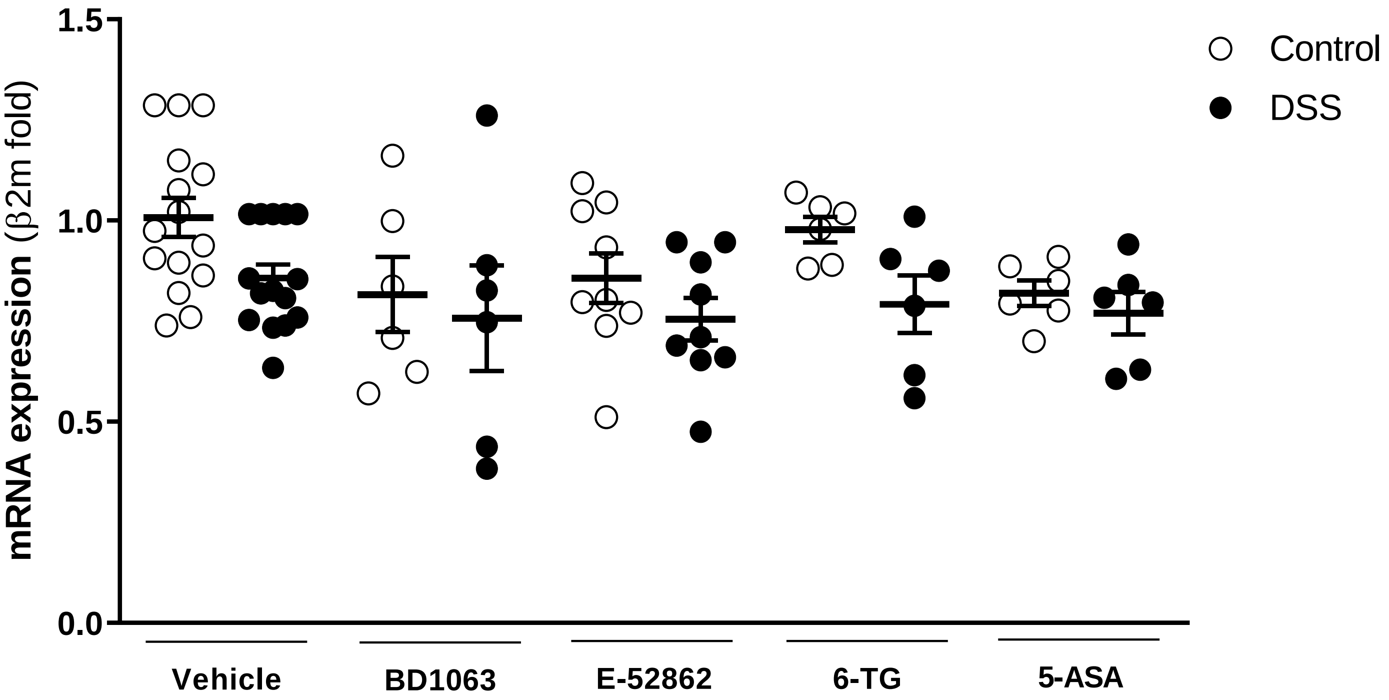

Supplement: Supplementary file 1 [file biomedicines-11-02758-s001.zip › biomedicines-2611087-supplementary.pdf]
